# Fungi in Mycelium-Based Composites: Usage and Recommendations

**DOI:** 10.3390/ma15186283

**Published:** 2022-09-09

**Authors:** Maciej Sydor, Grzegorz Cofta, Beata Doczekalska, Agata Bonenberg

**Affiliations:** 1 Department of Woodworking and Fundamentals of Machine Design, Faculty of Forestry and Wood Technology, Poznań University of Life Sciences, 60-637 Poznań, Poland; 2 Department of Chemical Wood Technology, Faculty of Forestry and Wood Technology, Poznań University of Life Sciences, 60-637 Poznań, Poland; 3 Institute of Interior Design and Industrial Design, Faculty of Architecture, Poznan University of Technology, 60-965 Poznań, Poland

**Keywords:** mycelium, fungi, biomaterial, bio-composite, bio design, mycelium-based material, mycelium-based composites, biopolymers, wood

## Abstract

Mycelium-Based Composites (MBCs) are innovative engineering materials made from lignocellulosic by-products bonded with fungal mycelium. While some performance characteristics of MBCs are inferior to those of currently used engineering materials, these composites nevertheless prove to be superior in ecological aspects. Improving the properties of MBCs may be achieved using an adequate substrate type, fungus species, and manufacturing technology. This article presents scientifically verified guiding principles for choosing a fungus species to obtain the desired effect. This aim was realized based on analyses of scientific articles concerning MBCs, mycological literature, and patent documents. Based on these analyses, over 70 fungi species used to manufacture MBC have been identified and the most commonly used combinations of fungi species-substrate-manufacturing technology are presented. The main result of this review was to demonstrate the characteristics of the fungi considered optimal in terms of the resulting engineering material properties. Thus, a list of the 11 main fungus characteristics that increase the effectiveness in the engineering material formation include: rapid hyphae growth, high virulence, dimitic or trimitic hyphal system, white rot decay type, high versatility in nutrition, high tolerance to a substrate, environmental parameters, susceptibility to readily controlled factors, easy to deactivate, saprophytic, non-mycotoxic, and capability to biosynthesize natural active substances. An additional analysis result is a list of the names of fungus species, the types of substrates used, the applications of the material produced, and the main findings reported in the scientific literature.

## 1. Introduction

**Figure 1:**
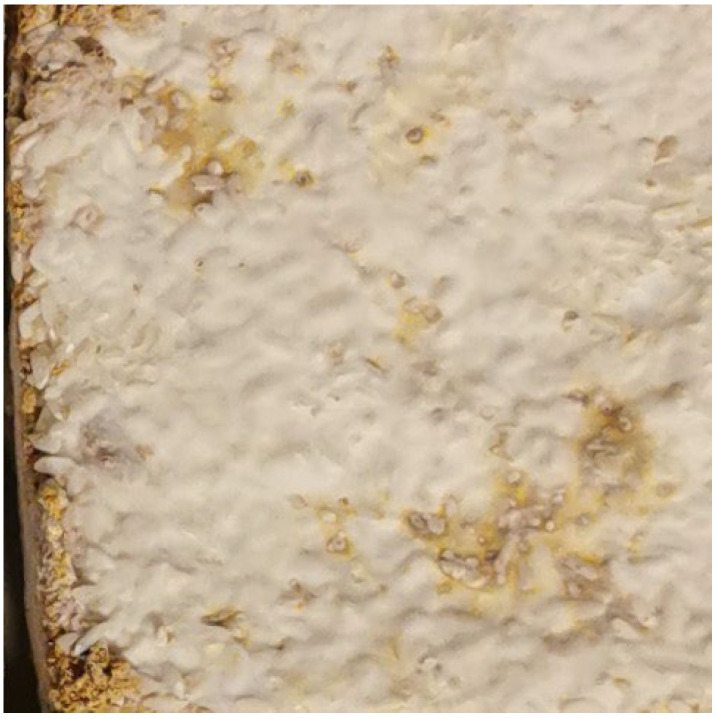
Sample based on hemp mix with rice. Surface: smooth, with grains of rice and substrate fibers; color: off-white, with irregularities (photo A. Bonenberg).

**Figure 2:**
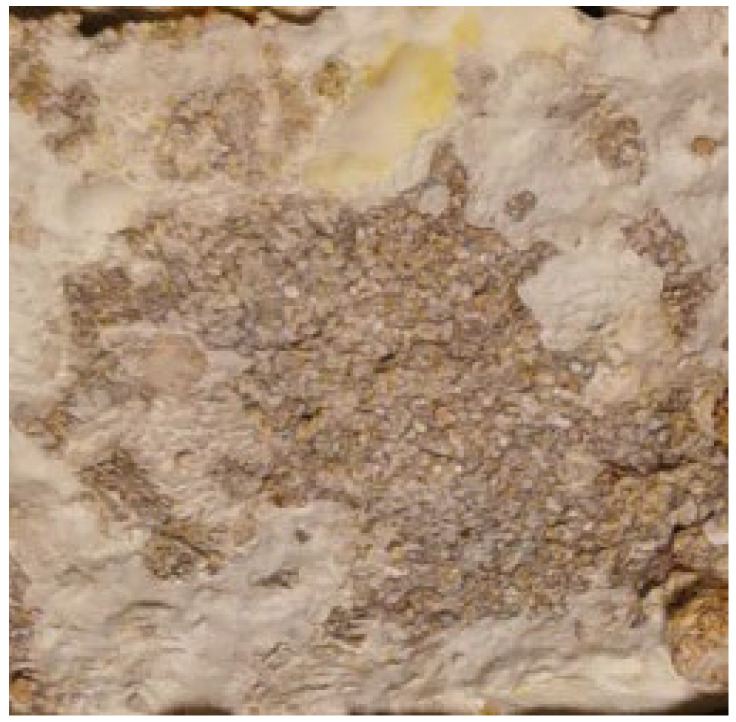
Sample based on hemp mix with buckwheat. Surface: rough, visible substrate fibers; color: two shades of gray (photo A. Bonenberg).

**Figure 3:**
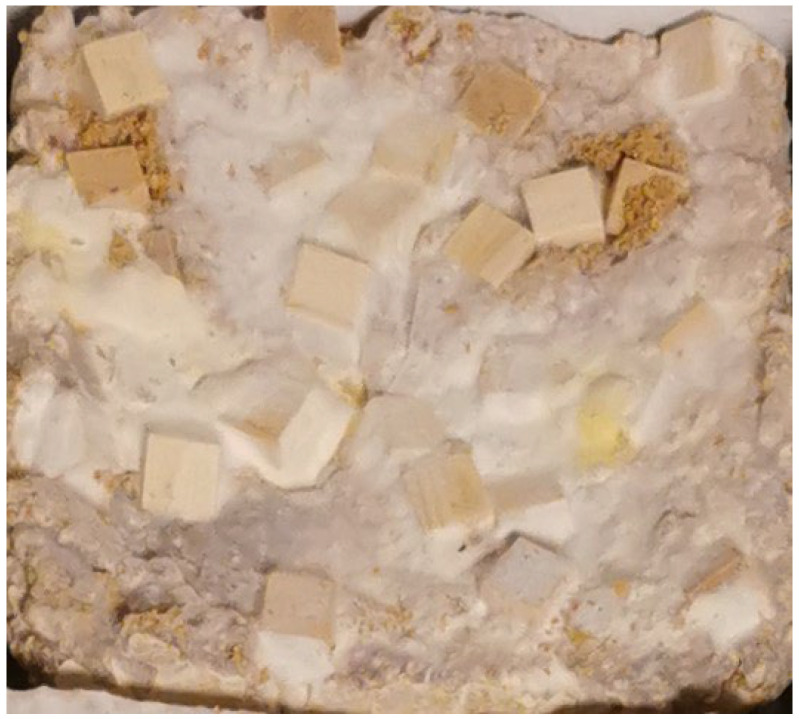
Sample based on hemp mix with wood cubes. Surface: smooth with inclusions; color: off-white, wood inclusions (photo A. Bonenberg).

**Figure 4:**
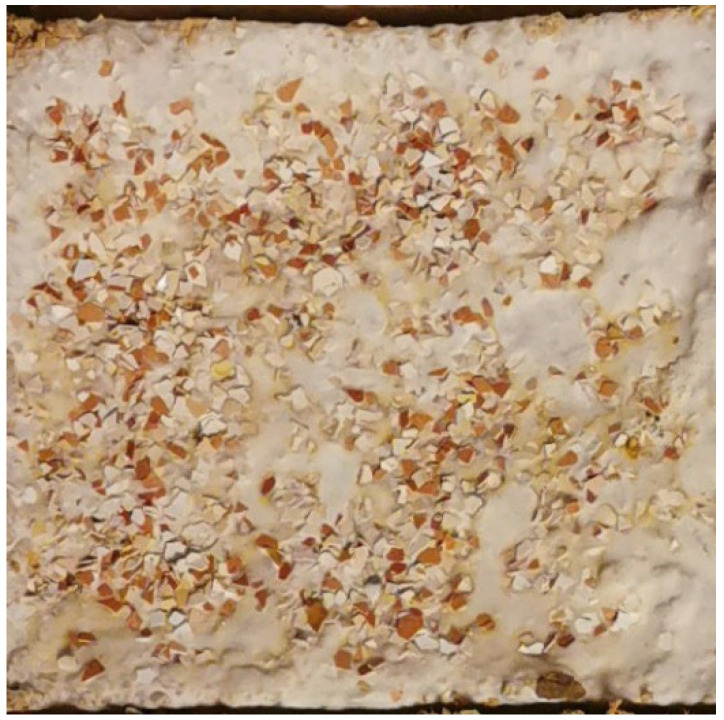
Sample based on hemp mix with eggshell. Surface: smooth with eggshell. Color: off-white mycelium, gray, eggshells (photo A. Bonenberg).

**Figure 5:**
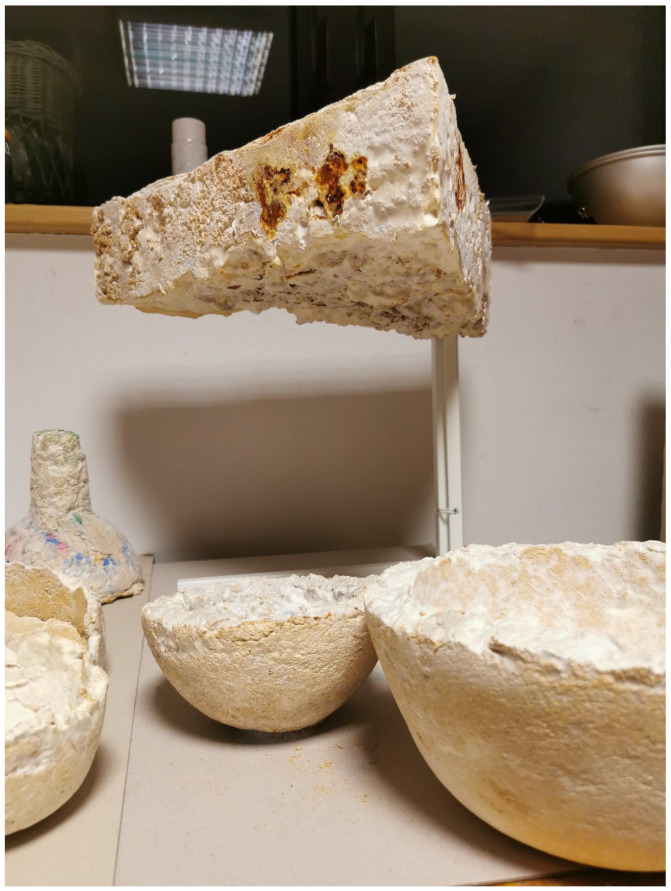
Various artefacts made with MBC: coffee-table, bowls, lampshades based on hemp mix with *Ganoderma lucidum* (artefact production and photo A. Bonenberg).

Mycelium-Based Composites (MBC) consist of defragmented lignocellulosic particles bonded with dense chitinous mycelium. These innovative biomaterials show eco-friendly characteristics: waste materials usage, low energy demand during production, the production does not generate waste, and the products are readily recycled [1]. The performance properties of MBC are usually inferior to those of the materials used so far. However, their advantages are revealed in some areas, such as high acoustic attenuation, fire resistance, the absence of harmful synthetic chemical components [2,3,4], and advantages connected with aesthetics. In turn, the drawbacks of MBC, which need to be eliminated, include excessive hygroscopicity, low tensile strength, susceptibility to biological corrosion, and the need to deactivate the fungus. Improving the properties of this innovative material is the goal of many scientific and commercial endeavors [5,6]. Thus, the potential applications of MBC may be found in architecture [7,8], packaging [9], the automotive industry [10], as a furniture material, in art [8], and in manufacturing various chitin- and β-glucan-based flexible materials, such as foams or paper, as well as textile substitutes [11]. The scientific literature describes the biocomposites as pure mycelium bio-materials, consisting only of mycelial biomass, e.g., myco-leather, as a substitute for petrochemically produced and animal-based leather [12]. There are also concepts for the use of mycelium to grow monolithic buildings from the functionalized fungal substrate [13] and as self-repairing wearable electronics, using various fungus properties (memristors, oscillators, pressure, and optical and chemical sensors) [14]. The results of our own feasibility studies on different surface structures of MBC required in art and architecture uses are shown in Figure 1, Figure 2, Figure 3, Figure 4 and Figure 5. In all cases, the *Ganoderma lucidum* was the binding agent, the substrates contained admixtures.

**Figure 6:**
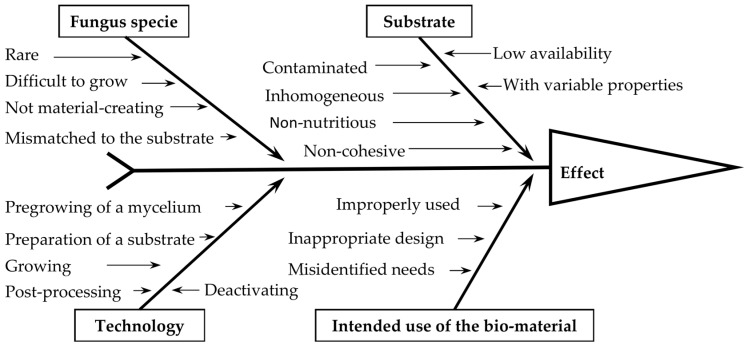
Factors affecting the manufacture and use of Mycelium-Based Composites.

The practical difficulty in the production of MBC is connected with an appropriate selection of the fungi species, substrate, and production technology. The produced MBC should be technologically feasible, profitable, provide expected physical properties in the entire volume, and be acceptable for humans. Difficulties in the appropriate selection of the fungi species result from the large variety of fungus species and available substrates, problems associated with combining a specific fungi species with a specific substrate in terms of mycelium growth, and inactivation parameters and different requirements for biocomposites [15]. Many fungi form mycotoxins, attract insects, or become invasive species [16]. The factors that may cause the biocomposite properties to differ from expectations are shown in Figure 6.

Current mycelium-based engineering materials are innovative with many advantages but have some disadvantages. The selection of an appropriate species of fungus for the substrate or the use of species that have not been used so far could eliminate these disadvantages. This choice could be adequately supported by the quantitative analysis of the fungi species described in the scientific documents to create a biocomposite with expected properties. There are no review articles comparing the intensity of studies of individual species of fungi and analyzing the most common combinations of fungus species–substrate. As is known, there are millions of fungi species, but only a few dozen are used to produce biomaterials. Furthermore, no general guidelines have been formulated in the literature to find new species to create mycelium-based materials. This review fills the research gaps in this regard. The present review is expected to contribute to discovering optimal combinations of species of fungus–substrate based on current research. The review also aims to propose scientifically justified criteria to be met by a newly used fungus specie to make available the optimal production of Mycelium-Based Composites.

## 2. Fungus Species in the Scientific Literature

Fungi are a group of organisms classified into separate kingdom. Defining characteristics include the presence of chitin in their cell walls, heterotrophism, and cosmopolitism [17]. The total number of fungi species is not known. To date, as few as approx. 150,000 species [18] have been described from the estimated number of 1.5 million up to 5.1 million species [19]. Commonly used databases containing updated information on fungi are Species Fungorum (www.speciesfungorum.org, Centre for Agriculture and Bioscience International (CABI), Wallingford, Oxfordshire, UK, accessed on 8 August 2022), and MycoBank (www.mycobank.org, Westerdijk Fungal Biodiversity Institute, Utrecht, Belgium, accessed on 8 August 2022). Fungi are classified using a phylogenetic tree [20], which orders these organisms into hierarchic groups. In nature, fungi are associated with other organisms through symbiosis and commensalism as parasites or reducers. Considering these dependencies, fungi are classified as harmful (causing disease or depreciation) or beneficial organisms (mycorrhizas).

From 2012 to 2022, almost 100 original articles were published [21,22,23,24,25,26,27,28,29,30,31,32,33,34,35,36,37,38,39,40,41,42,43,44,45,46,47,48,49,50,51,52,53,54,55,56,57,58,59,60,61,62,63,64,65,66,67,68,69,70,71,72,73,74,75,76,77,78,79,80,81,82,83,84,85,86,87,88,89,90,91,92,93,94,95,96,97,98,99,100,101,102,103,104,105,106,107,108,109,110,111,112,113], presenting almost 70 species of fungi used to produce Mycelium-Based Composites; these species are listed in Table 1. The growth conditions used in the cited studies, inactivation methods, and the results achieved are listed in Appendix A.

**Figure 7:**
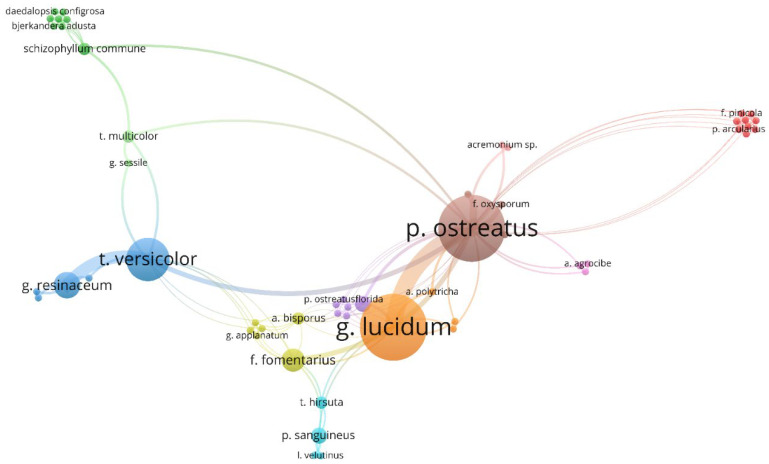
Fungus species in the scientific literature related to Mycelium-Based Composites.

As can be seen from Table 1, most studies on Mycelium-Based Composites concern white rot fungi. Some scientific publications describe the results of comparative analyses for various fungus species. The visualization of the frequency of research and the frequency of scientific comparisons of different species of fungi is given in Figure 7. The size of the circle shows the popularity of the fungus species in the scientific literature and the lines indicate the most frequently used comparisons of the fungus species in scientific publications.

It results from Figure 7 that two fungus, *Pleurotus ostreatus* (mentioned in 22 documents) and *Ganoderma lucidum* (mentioned in 20 documents), are most frequently mentioned in scientific publications. Another commonly used species is *Trametes versicolor* (10 times). *P. ostreatus* and *G. lucidum* are the most frequently compared species. All these species cause white rot. A detailed list of fungus species, substrates, technological parameters, research aims, and main findings based on almost 100 original articles is given in Appendix A.

## 3. Fungus Species in Patent Documents

There are several hundred patent documents concerning Mycelium-Based Materials [8]. The oldest document was filed at the United States Patent and Trademark Office on 12 December 2007 [114]. Patent documents mention several dozen fungus species. They are listed in Table 2, giving the specie names, the number of patent documents specifying a given species or family, and references to the first patent document in which this species was mentioned.

It is worth highlighting that patent documents do not provide detailed knowledge concerning the effectiveness of the mentioned fungus species, as is typically seen in scientific documents. Admittedly, all patent documents disclose the essence of the invention, but conversely, providing too much information is clearly against the interests of the owner of the invention. For this reason, patent documents contain a minimum of knowledge and simultaneously make producing a similar solution as complicated as possible [125].

## 4. Substrate Type Analysis

**Figure 8:**
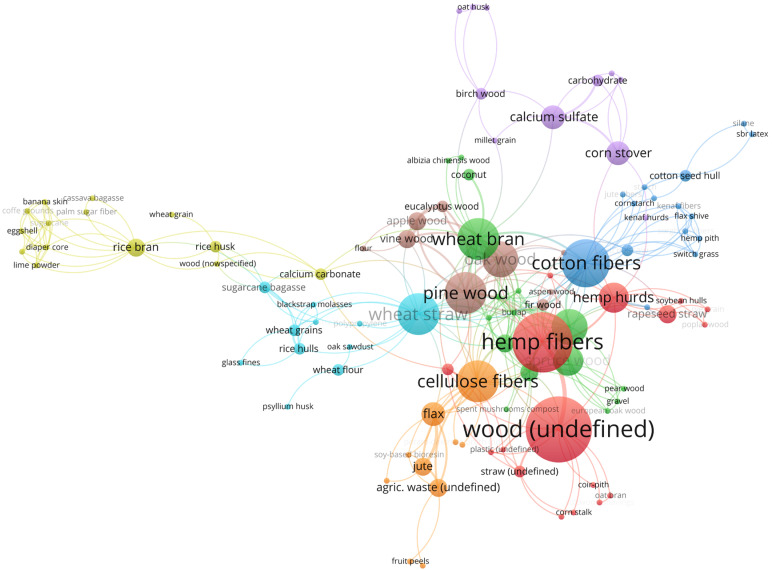
Combinations of substrates used in scientific experiments.

Substrates for the production of Mycelium-Based Composites originate from three sources: agricultural by-products, industrial waste, and post-consumer waste. In terms of their composition, these substrates can be divided into annual plants, softwood, and hardwood. Common bulk substrates include several components: wood chips or sawdust, mulched straws (wheat, rice, and others), chopped corncobs, recycled paper, nut and seed hulls or meal, coffee pulp or grounds, and brewer’s grain. An ideal substrate contains enough nitrogen and carbohydrates for rapid fungal mycelium growth. Various substrates are compared in scientific analyses or combined as mixtures in different proportions. Combinations of various substrates in scientific experiments, described in 85 scientific publications [21,23,24,25,26,27,28,29,30,31,32,33,34,35,36,37,38,40,41,42,45,46,49,50,51,52,53,57,58,59,60,61,62,63,64,65,66,67,68,70,72,73,74,75,76,77,78,79,80,81,82,83,84,86,87,88,89,90,91,92,93,94,95,96,97,98,99,100,101,102,103,104,105,106,107,108,109,111,112], are presented in Figure 8. The size of the circle shows the popularity of the substrate and the lines indicate the most frequently used comparisons of substrates in scientific publications.

It can be seen from Figure 8 that pine wood is the wood material most commonly used as a substrate. In turn, fibrous plants with high cellulose contents, i.e., hemp, cotton, and wheat straw, were the most frequently used among annual plants. In terms of the expected strength, the substrate materials should be long (strand type), thus wood chips and straw are preferred. Regarding technological requirements, an abundant and uniform supply of substrate materials is needed, while low acquisition cost is essential in the production economy.

All these raw materials are lignocellulose materials. They are composed of 30–50% cellulose, 15–30% lignin, and 25–35% hemicelluloses as well as non-structural substances (e.g., pectins, waxes, pigments, tannins, lipids, and minerals). Their composition is dependent on their origin and species [126,127,128]. Cellulose is the primary structural component of all plant fibers [129]. It is a natural polymer. Cellulose molecules consist of glucose units linked together in long chains (β-1,4 glycoside linkages join the repeating units of D-anhydro glucose C_6_H_11_O_5_), which in turn are linked together in bundles called microfibrils. This principal component provides them with strength, stiffness, and stability. Hemicelluloses are polysaccharides bonded together in relatively short, branching chains. They are closely associated with cellulose microfibrils, embedding cellulose in a matrix. Hemicelluloses are highly hydrophilic. The molecular weights of hemicelluloses are lower than that of cellulose. Lignin is a complex aromatic hydrocarbon polymer that imparts rigidity to plants. Without lignin, plants could not attain great heights. Lignin is a three-dimensional polymer with an amorphous structure and a high molecular weight, and it is less polar than cellulose. It serves as a chemical adhesive within and between fibers. Lignin acts primarily as a structural component by adding strength and rigidity to the cell walls. However, it also allows the transport of water and solutes through the vascular system of plants and provides physical barriers against invasions of phytopathogens and other environmental stresses. It consists of three basic phenylpropanoic monomers known as monolignols: p-coumaryl, coniferyl, and sinapyl alcohols [130].

When incorporated into the lignin polymer, the units that originated from the monolignols are called p-hydroxyphenyl (H), guaiacyl (G) and syringyl (S) units, respectively. The amount of lignin varies according to the origin of the lignocellulosic starting material. At the same time, the proportion of different monolignols and chemical bonds in the lignin structure also depends on the lignocellulosic biomass, because these vary between hardwood, softwood, or grass. In softwoods, lignin is mainly composed of guaiacyl units linked by ether and carbon-carbon bonds, whereas in hardwoods, lignin has equal amounts of guaiacyl and syringyl units. The grass lignin is characterized by guaiacyl, syringyl, and hydroxyphenyl units.

Several methods are employed to sterilize the substrate, thereby rendering the substrate inert. This can be provided (1) by temperature, i.e., heat treatment, such as autoclaving and pasteurization, or (2) treatment with chemical or microbial agents (Appendix A). Sterilization in an autoclave is typically run at temperatures ranging from 115 to 121 °C for 15 to 120 min. In turn, the pasteurization is run in water at a temperature of 100 °C for approx. 100 min. Substrates may also be subjected to the action of a hydrogen peroxide solution at a concentration ranging from 0.3% to 10%.

**Figure 9:**
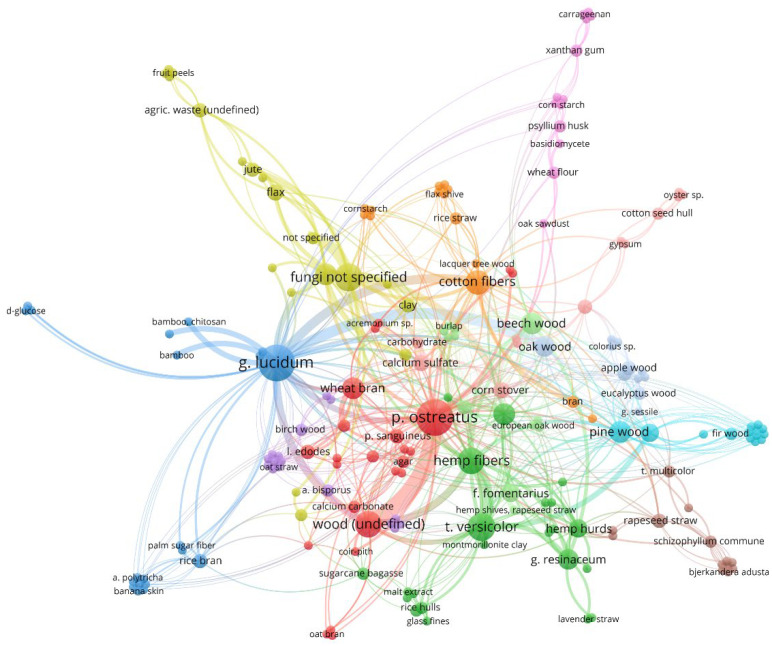
Combinations of substrates and fungus species used in scientific experiments.

The substrate has to contain the nutrients required for fungus growth to improve the growth rate and modify mechanical strength properties, which the mycelium matrix attains after growth. Simple sugars, such as glucose, are used as additives. The addition of glucose to the lignocellulose material results in the lesser degradation of holocellulose at the preliminary stage of degradation caused by fungi. Figure 9 illustrates lignocellulose substrates linked with various fungus species in original articles related to Mycelium-based Composites [21,23,24,25,26,27,28,29,30,31,32,33,34,35,36,37,38,40,41,42,45,46,49,50,51,52,53,57,58,59,60,61,62,63,64,65,66,67,68,70,72,73,74,75,76,77,78,79,80,81,82,83,84,86,87,88,89,90,91,92,93,94,95,96,97,98,99,100,101,102,103,104,105,106,107,108,109,111,112]. As with Figure 7 and Figure 8, the size of the circle shows the popularity of the mushroom species or substrate, and the lines indicate the most common combinations of fungal species and substrates in scientific publications.

Substrates derived from both hardwood and softwood materials were typically combined with white rot fungi, i.e., *T. versicolor* and *P. ostreatus*. Additionally, composites based on fibrous plants were obtained mainly using white rot fungi *T. versicolor*, *P. ostreatus*, and *G. lucidum*. The critical observation is that the white rot fungi can degrade lignin in the plant cellwall by skipping cellulose, unlike the other wood degrading fungi. The following patterns have been found when studying lignin bioconversion by basidiomycetes: (1) the first stages include lignin demethoxylation and subsequent hydroxylation, which is accompanied by a decrease in the number of methoxy groups and an increase in hydroxyl groups; (2) then the αC–βC bond is broken with oxidation of the first hydroxyl to carboxyl group; and (3) the aromatic ring in lignin is broken [131].

Following mycelium growth, the resulting composite materials may be removed from molds and hot pressed, dried in an oven or air-dried to dehydrate the obtained material and neutralize the fungus. Consequently, fungi may no longer grow or spread while the composite material is rigidified. Hot pressing and oven drying are preferred treatment methods in industrial practice because they are the fastest dehydration methods. As a result of hot pressing, the material is consolidated and condensed, which results in higher values of mechanical strength properties.

A systematic review of applied MBC growth parameters is given in Appendix A.

## 5. Discussion: Fungus Species Recommendations

The main purpose of the literature review described in this article is to indicate attributes of an ideal fungus species to create mycelium-based materials. The 11 such attributes have been identified: (1) rapid growth of hyphae, (2) high virulence, (3) dimitic or trimitic hyphal structures, (4) white rot fungi, (5) high versatility in nutrition, (6) tolerance to a wide range of substrate parameters and environmental conditions, (7) susceptible to readily controlled ecological factors, (8) easy to deactivate, (9) saprophytic, (10) non-mycotoxic, and (11) having the ability to biosynthesis natural active substances.

(1–2) A Mycelium-based Composite (MBC) for engineering usage needs to exhibit isotropic physical properties, thus an optimal organism should bind the organic matrix into a composite with such properties. In this case, it is best to select an appropriate organism assuming the division proposed by Harper [132] into modular and autonomous organisms. Modular organisms develop through repeated iterations of modules, and such a repeatable structure facilitates the exploitation of a static resource (substrate) by an immobile organism (a fungus). Mycelium hyphae absorb nutrients serving the role of building components in the area where they are growing. A solution to the problem of nutrient depletion around hyphae is offered by the regrowth of hyphae from the depleted substrate zone at the simultaneous production of successive modules, i.e., hyphae located so that the zones are devoid of nutrients do not overlap. Harper showed a lack of mutual overgrowth of young hyphae. This mechanism ensures the rapid colonization of large substrate areas acting as a matrix in the biocomposite. Modular organisms may find food using two strategies: guerrilla and phalanx. Fungi degrading lignocellulose materials find food using the phalanx method. This phalanx growth type involves extensively branched hyphae facilitating colonization of such a substrate. This type of growth is observed in white rot fungi. Hyphae produce high local concentrations of extracellular enzymes and other chemical substances, preventing the colonization of the substrate by other organisms. This mechanism supports the axenic culture during biocomposite production. The hyphae’s anatomy and the modular structure ensures fungal survival in the case of mechanical damage to the mycelium. Internal organelles, such as Woronin’s bodies, can plug the septal pores to prevent cytoplasm loss from hyphae. Every single hypha may reproduce, forming another organism. This property makes it possible to obtain large amounts of the material used as an inoculum within a short time.

Hyphae can regrow from the substrate, facilitating their transition through the gas phase to penetrate new sites abundant in nutrients. This is because most currently known fungi are organisms living in the terrestrial environment with the predominance of the gas phase over the liquid phase. This property considerably facilitates the colonization of a loose lignocellulose material. As it results from the above, the fungal mycelium seems to be the most adequate for biocomposite production among all the organisms colonizing our planet. More details concerning the modular structure of mycelium may be found in a publication by Calile [133].

The rapid growth of hyphae, combined with the possibility to initiate the development of new mycelium by its fragment, makes it possible to obtain large amounts of inoculum within a short time. Increased inoculum density in the substrate results in a reduction in lag phase time, increased specific growth rate, improved maximum efficiency, and lowered substrate degradation. Jones et al. [134] were of the opinion that the optimum inoculation density is 10–32% inoculum to substrate ratio (by volume) depending on the used inoculum, whether in the liquid or solid form. In terms of the efficient formation of the biocomposite, it is desirable to minimize the lag phase and provide optimal environmental conditions and abundance of nutrients to maximize growth rate and efficiency and prevent the premature transition to the stationary growth phase.

An isotropic composite has to be manufactured under sterile conditions, which is required for the rapid and uniform colonization of the substrate in the axenic culture (monoculture). This increases the chance of obtaining a material exhibiting comparable properties over the entire material volume. In the case of incomplete substrate sterilization, the produced biocomposite may exhibit various physical properties differing from those assumed [135]. The colonization of dead wood by fungi under natural conditions takes the form of microbial succession. Wood is first colonized by rapidly growing more primitive microorganisms (e.g., mitosporic fungi), which are next replaced by higher fungi (white, brown, and grey rot fungi). This is not an absolute requirement, but it depends rather on the local conditions and present fungal strains. In the case of axenic cultures in mycelium-based composite formation, we need to consider the phenomenon of the succession of microorganisms colonizing the substrate. The division into three groups based on the colonization rate of all substances also needs to be remembered. Primary colonizers appear as the first microorganisms. A rapid growth rate characterizes them; they spread fast and degrade simple compounds. Secondary colonizers rely on primary colonizers, which partially degrade the organic matter before digestion of more complex compounds. Tertiary colonizers appear towards the end of the degradation process, taking advantage of the conditions created by primary and secondary colonizers. When the dependencies mentioned earlier are not considered, the substrate colonization rate by the fungus used to produce the biocomposite may be slower than initially assumed. This harms the economic aspect of biocomposite manufacture.

(3) When considering fungus species for producing biomaterials, the species producing leathery or woody fruiting bodies should be considered. They have a complex system of dimitic and trimitic hyphae. The function of fungal hyphae is to bind the biocomposite matrix. This is achieved most effectively by dimitic or trimitic hyphae, providing mycelium with better physical properties than the mycelium containing only generative hyphae (monomitic fungi). Apart from the generation of a branched network structure, the increased contact area with the composite matrix hyphae should contain adhesive substances, such as hydrophobins.

(4) Fungi used to produce biocomposites need to cause a simultaneous white rot of the substrate. Regarding MBC strength, the cellulose in the substrate must remain undegraded, therefore selective white rot is the preferred type of degradation during mycelial growth. The selection of a fungus causing this type of degradation prevents the defibration of wood during degradation even in a highly advanced process [136]. White rot fungi causes xylem defibration, which will provide a composite with poorer physical parameters. Fungi have to degrade lignin more effectively than holocellulose, thanks to which better physical properties of the substrate are maintained, compared to fungi causing brown or grey rot [137]. White rot fungi cause a uniform volumetric shrinkage of the isotropic substrate, observable only when the loss of substrate mass exceeds 40–50% [138]. It minimizes volumetric changes in the composite matrix during the production process. This is also reflected in the compressive strength of MBC, which is dependent on the substrate structure (matrix). In the case of fungi causing brown or grey rot, the volumetric changes of wood are anisotropic and found at a much earlier stage of degradation. Brown and grey rot fungi cause an adverse loss of holocellulose, so the composite matrix has much poorer physical parameters than the original parameters of wood.

(5) Optimal fungi for composite production must colonize and degrade many different lignocellulose materials and other waste generated by the agricultural, forestry, and food industries. Moreover, these fungus species should biodegrade various synthetic chemical substances providing a wider range of potential substrate types to manufacture composites. This makes it possible to use lignocellulose matrices contaminated with other substances.

(6) The properties of Mycelium-Based Composites are significantly affected by their production parameters, such as growth time and conditions, incubation temperature, the pH and moisture content of the substrate, access to light, and the material drying methods. These parameters vary for different fungal strains and used substrates. Manufacturing parameters may be modified to influence the properties of produced biocomposites. Incubation time depends on substrate volume and ranges from 5 to 42 days depending on the fungus species. The optimal incubation temperature ranges from 21 to 30 °C for different fungus species.

(7) Similarly, the substrate pH level for optimal growth in the case of various fungi ranges from 5 to 8, while humidity from 80 up to 100%. Because of biocomposite production, the used fungal species have to be readily maintained in the anamorphic stage, not producing fruiting bodies. This process may be controlled using CO_2_ concentration, elevated temperature (30–35 °C), and lack of access to light. It results from the above that the fungus species should be thermophilic and tolerate the CO_2_ content in the culture chamber atmosphere.

**Figure 10:**
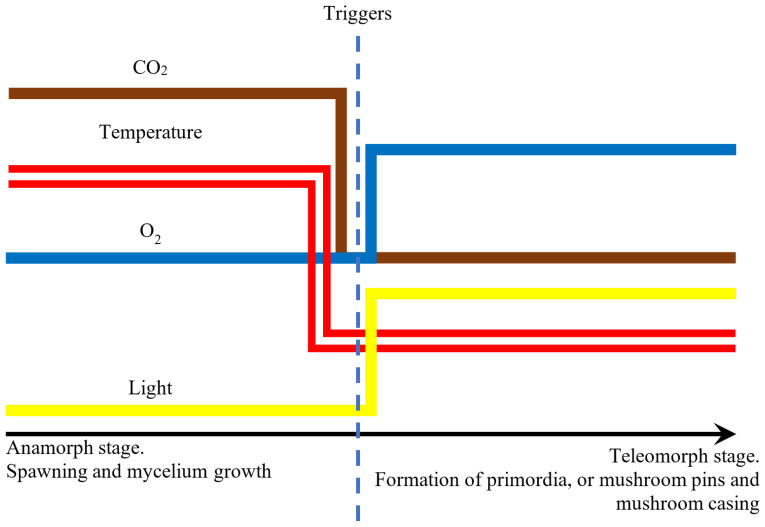
Triggers for the growth of fruiting bodies.

Preferential conditions for the production of a biomaterial with high mycelium density include a lack of light radiation, increased carbon dioxide concentration at a simultaneous reduced oxygen concentration, and elevated temperature (18–35 °C). Figure 10 presents parameters causing changes in the fungus development stage.

(8) Mycelium deactivation in MBCs may consist in heat denaturation or otherwise. The heat denaturation requires the element made from an MBC to be placed in a drier. To improve an economic efficiency of biocomposite production, the mycelium should be deactivated at the lowest possible temperature, e.g., 60 °C, or applying other safe and, at the same time economically viable methods, e.g., microwave radiation. This can facilitate the deactivation process and provide the deactivation of large-sized elements.

(9) Fungi used in biocomposite production have to be saprophytic, not parasitic, since the latter are frequently pathogenic. Using saprophytes to produce biocomposites will reduce health hazards for humans and other organisms, particularly homeothermic animals, in the case of an uncontrolled release of biomaterials to the natural environment. Such a situation may occur when no effective deactivation is performed following the culture process. 

(10) An important feature of pathogenic fungi is connected with the synthesis of mycotoxins and microbial volatile organic compounds (MVOC). These substances of natural origin very often cause various diseases in humans.

(11) For this reason, it seems advisable to consider either medicinal effects or the neutral effect on the homeothermic organisms in the course of production and the use of biocomposites. This will reduce the risk during composite production and use in environmental protection aspects. It may even reduce manufacturing costs thanks to the production of biologically active substances, such as medications. Secondary fungal metabolites, which exhibit antimicrobial action, may be applied in materials used in the food industry. The biocomposite obtained using mycelium synthesizing active substances may have contact with food if the used organism is edible and free from toxic substances. Such properties may be found in edible mushrooms and fungi used in natural medicine to provide medicinal substances.

## 6. Summary and Conclusions

The most important reasons for using Mycelium-Based Biocomposites (MBC) include the management of by-products, the storage of carbon dioxide from the atmosphere, reduced need for petrochemicals in produced materials, and recyclability as well as interesting aesthetic features. Substrates for the manufacture of MBCs come from three primary sources: agricultural by-products, industrial waste, and post-consumer waste. In the case of substrates for industrial MBC production, it is vital to ensure their constant, abundant supply and availability. The functional properties of MBC are usually inferior to those of the materials used currently; however, in some areas, advantages of this innovative material need to be stressed, such as high acoustic attenuation, fire resistance, absence of chemicals, and, finally, aesthetic features, even though the latter is difficult to parameterize. The appropriate selection of the fungus species for the substrate is key to achieving the expected MBC properties. Millions of fungus species are still unknown to science, thus providing an excellent opportunity to identify fungi capable of producing MBCs with even better characteristics. Based on the analysis of many literature sources, 11 features were formulated to increase the effectiveness of fungi in the manufacture of MBC:

1. **Rapid linear growth of hyphae** will facilitate the production of large amounts of inoculum in a short time and will contribute to the minimization of the biocomposite production time. Moreover, the substrate will not be excessively degraded by mycelium.
2. **High virulence**. Fungi must be able to rapidly colonize the substrate before other microorganisms do. The aim is to obtain an axenic, uniform, dense fungal culture in the substrate. Thus, a biocomposite with isotropic physical properties is obtained.
3. **Hyphal structures**. The hyphae of the fungus, which provide a lattice for biocomposites, should be dimitic or trimitic, thus producing mycelium with better strength properties than the mycelium containing only generative hyphae (monomitic fungi). For this reason, the mycelia of mushrooms with hard leathery or woody fruiting bodies need to be used because they form mainly dimitic and trimitic hyphae.
4. **White rot fungi**. Fungi that cause white rot are preferred. These fungi degrade lignin in the cell walls of woody plants to a greater degree than they do with cellulose—thus, the composite matric has better physical parameters compared to the application of brown rot or grey rot fungi.
5. **High versatility in nutrition.** The fungus used in MBC needs to grow on a wide range of lignocellulose materials and on many other materials, e.g., plastics. The availability of various substrates will reduce the manufacturing costs of materials.
6. **High tolerance to a wide range of substrate parameters and environmental conditions**. Selected fungal specie should exhibit high tolerance to various environmental conditions, i.e., temperature and humidity, as well as the analogous parameters of the substrate, including non-uniform substrate moisture content and pH. This can simplify an MBC manufacturing technology.
7. **Fungi susceptible to readily controlled ecological factors**, such as temperature, light intensity, carbon dioxide concentration, oxygen concentration, or other technological factors. These parameters may promote the rapid linear growth of hyphae while preventing the formation of fruiting bodies.
8. **Mycelium easy to deactivate**. Mycelium in an MBC should be susceptible to deactivation using various methods. This will enable the production of large MBC elements and increase the human acceptance level of manufactured MBC products.
9. **Saprophytic fungi**. Fungi for the production of MBC may not be facultative parasites, since otherwise, the produced biocomposite may be hazardous for humans.
10. **Non-mycotoxic fungi**. The fungus should not synthesize harmful metabolites, e.g., mycotoxins or microbial volatile organic compounds (mVOC). Mycotoxins and mVOC may cause disease or even death in humans and other animals.
11. **The biosynthesis of natural active substances**. Fungi preferred in the production of biocomposites might synthesize natural active substances. This will reduce production costs and provide biocomposites with unique properties.

## Figures and Tables

**Table 1:** Fungus species in scientific publications related to Mycelium-Based Composites.

Decay Type	Fungus Species and Literature References
Brown rot	*Fomitopsispinicola* [63]; *Gloeophyllum sepiarium* [63]; *Laetiporus* *sulphureus* [63]; *Phaeolus schweinitzii* [63];
Soft rot	*Acremonium* sp. [96]; *Fusarium oxysporum* [94]; *Oudemansiella* *radicata* [96]; *Trichoderma asperellum* [77], *T. asperellum* [77];
White rot	*Agaricus bisporus* [59,77,87]; *Auricularia polytricha* [81]; *Ceriporia lacerata* [30]; *Colorius* sp. [61]; *Cyclocybe aegerita* (specified as *Aaegerita agrocibe*) [36]; *Coprinopsis cinerea* [62]; *Daedaleopsis confragosa* [44]; *Flammulina velutipes* [77]; *Fomes fomentarius* [38,83,85,87,108]; *Fomitopsis pinicola* [63]; “*Ganoderma* sp.” [21,41,44,61,68,77,110], *G. applanatum* [87], *G. boninense* [75], *G. lucidum* [22,25,31,32,41,69,70,72,73,74,77,79,80,81,82,83,89,100,102,106,109,112], *G. resinaceum* [44,49,86,93,101], *G. sessile* [61,110]; *Inonotus obliquus* [67]; *Irpex lacteus* [42]; *Kuehneromyces mutabilis* [77]; *Laetiporus sulphureus* [63]; *Lentinula edodes* [32,64,77]; *Lentinus velutinus* [67]; *Megasporaporia minor* [49]; *Oxyporus* *latermarginatus* [49]; *Phaeolus schweinitzii* [63]; *Piptoporus* *betulinus* [63]; “*Pleurotus* sp.” [33], *P. albidus* [67], *P. citrinopileatus* [74], *P. djamor* [62], *P. eryngii* [74], *P. ostreatus* [26,29,32,35,36,37,38,41,46,56,57,63,74,77,81,82,84,88,90,91,94,96,99,103,105,106], *P. ostraceus florida* [77], *P. ostraceus sajorcaju caju* [77], *P. salmoneo-stramineus* [36]; *Polyporus* *arcularius* [63], *P. brumalis* [59], *P. pulmonarius* [36]; *Pycnoporus sanguineus* [67,83,92]; *Trametes* sp. [53,61]; *Trametes hirsuta* [83,99,104], *T. multicolor* [46,57,110], *T. pubescens* [63], *T. suaveolens* [63], *T. versicolor* [29,36,44,50,59,65,66,78,86,87,101,110], *Trichaptum* *abietinu* [63]; *Schizophyllum commune* [46,48,53,57]; “white-rot saprotrophic fungi, endemic to Alaska” [42]
Probably white rot	Specified as “*phylum Basidiomycetes*” [24,40,51]

**Table 2:** Fungus species in patent documents.

Division	Order	Fungus Species	No. of Patent Documents	Ref. to the Oldest Patent Document
*Basidiomycota*	*Agaricales*	*Agaricus* sp.	19	[114]
		*Agrocybe* sp./*Agrocybe aegerita*/*A. brasiliensis*	6/13/10	[115]/[116]/[114]
		*Coprinus comatus*	24	[114]
		*Flammulina velutipes*	13	[114]
		*Hypsizygus* sp. (as “*Hypsizygous* sp.”)	2	[115]
		*Hypholoma capnoides/H. sublaterium*	11/10	[114]/[114]
		*Lentinula edodes*	17	[116]
		*Macrolepiota procera*	11	[114]
		*Omphalotus* sp.	2	[115]
		*Pleurotus djamor*/*P. eryngii*/*P. ostreatus* var. *columbines*/*P. ostreatus*	16/15/13/45	[116]/[116]/[116]/[114]
		*Schizophyllum* sp.	14	[117]
	*Hymenochaetales*	*Inonotus obliquus*	24	[114]
	*Polyporales*	*Ceriporiopsis* sp.	2	[115]
		*Fomes fomentarius*	13	[118]
		*Ganoderma appalantum*/*G. lucidum* (also as “*lucidem*”)/*G. oregonense*/*G. resinaceum*, *G. tsugae*	3/42/23/11/27	[118]/[118]/[116]/[119]/[114]
		*Grifola frondosa*	15	[116]
		*Laetiporus* sp.	2	[115]
		*Phanerochaete* sp.	7	[117]
		*Piptoporous betulina* (as “*betulinus*”)	8	[120]
		*Polyporellus* sp.	2	[115]
		*Polyporus avleolaris*/*P. mylittae*/*P. squamosus*	3/3/8	[118]/[118]/[118]
		*Pycnoporus cinnabarinus*	4	[121]
		*Trametes versicolor*	19	[120]
	*Russulales*	*Hericium erinaceus*	4	[122]
*Ascomycota*	*Pezizales*	*Morchella angusticeps*	11	[114]
	*Xylariales*	*Xylaria polymorpha*/*X. hypoxylon*/*X. filiformis*/*X. longipes*	4/4/3/1	[117]/[117]/[117]/[123]
*Zygomycota*	n.d.	n.d.	1	[124]

## Data Availability

Not applicable.

## Appendix A

**Table A1:** A systematic review of fungus species, growth parameters, and main results in the scientific literature.

	Fungi	Substrate	Substrate Sterilization	Incubation and Growing	Denaturing and Drying	Product/Application	Results	Reference
1	*Ganoderma* sp.	Cotton-based (processed cotton carpel, cotton seed hull, starch, and gypsum)	115 °C, 28 min	21 °C, 5 days in the plastic mold shaped as the piece to be fabricated.	60 °C, 8 h	Packaging material	MBC meets or exceeds the characteristics of extruded polystyrene foam	[21]
2	*Ganoderma lucidum*	Red oak wood, nutrient solution (pending IP); 5.0 to 15 mm chips	Not specified	Not specified	220 °C, 120 min, from 60–65% to 10–20% MC, then seasoned to ca. 6% MC	Foam core of sandwich board	MBC is frangible resulting in a low ultimate tensile strength and a high stiffness. The strength of MBC increases with decreasing moisture content. The MBC has an average density and strength; its properties are closest to those of expanded polystyrene foam	[22]
3	Not specified (supplied by Ecovative Design)	Rice husk, wheat grain (three variants: 50/50, 70/30, 30/70)	In high pressure saturated steam, 121 °C, 15–20 min.	21 days in the container	50 °C, 46 h	Insulative packaging material	Comparing to polystyrene foam the MBC are 100% biodegradable, non-toxic, produce ten time less carbon dioxide (CO_2_) and require about eight times less energy to produce.	[23]
4	Not specified (probably as [21])	Rice straw, hemp pith, kenaf fiber, switch grass, sorghum fiber, cotton bur fiber and flax shive	Through the process reported by [21]	As [21]	As [21]	Insulation panels	Optimal performance at the noise frequency of 1000 Hz. MBC are comparable to polyurethane foam board and are better than plywood	[24]
5	*Ganoderma lucidum*	As [22]	Not specified	vacuum skin mold (bag)	As [22]	Foam core of sandwich board	The flexibility of layered structures depends on the technological parameters used.	[25]
6	Probably *Pleurotus ostreatus* (Oyster mushroom)	Cotton seed hulls, carboxylated styrene butadiene rubber (sbr) latex, and silane coupling agent	Not specified	5–7 days	Oven	The latex-mycelium composite insulation material	5% latex admixture increases the strength of the MBC, 10% latex kills the mycelium. Silane slightly increases strength, does not harm the mycelium	[26]
7	Not specified	Spent mushrooms compost (from 0% to 17%), clay	Compost oven-dried at 110 °C	No incubation	Not applicable	Brick (to build walls)	17% reduction in compost of 10% thermal transmittance	[27]
8	*T. versicolor*, *Pleurotus ostreatus*	Hemp (hurd/mat/fibers), wood chips (not specified)	Boiling 100 min. or 0.3% hydrogen-peroxide	Room temp., 90 to 100% RH, dark conditions, 30 days	Oven at 125 °C, 120 min.	Insulating foam	Hemp-mat + T. versicolor has the highest compressive strength	[29]
9	*Ceriporia lacerata*	Soybean straw	Without sterilization	25 °C for 5 days	Dried at 60 °C	Construction board	High compressive strength, good thermal insulation and good sound absorption	[30]
10	*Ganoderma lucidum*	Wood and additives (not specified)	Not specified	25 ± 3 °C, low light, 14 days	Above 70 °C, to 5% MH	Checking the susceptibility to machining	–	[31]
11	*Lentinula edodes*, *Pleurotus ostreatus*, *Ganoderma lucidum*	Straw, wood shavings, corn stalk, rice husks	hydrogen-peroxide	Under moist conditions in the dark, about 2 weeks	Not specified	Panels	Mold disinfection is crucial to avoid growth of any species other than the fungi	[32]
12	*Pleurotus* sp.	Crop residues, carrageenan, chitosan, xanthan gum	85 °C for 120 min	23 °C, 30 days, wooden molds	25 °C, 48 h	Packaging material	MBCs do not pose as an alternative to expanded polystyrene	[33]
13	Not specified	Core: agricultural waste substrates; outer layers: jute, flax, cellulose	10% hydrogen peroxide	Semi-permeable polypropylene bag, up to 98% RH, incubation process: 5 days, 24 °C.	A convection oven at 82 °C for 12 h and 93 °C for 8 h, pressed at 250 °C for 20 min	Packaging material	Flexural strength depends on the degree of colonization of the mycelium within the outer layers and the bonding between the outer layers and the core. Stiffness depends on the core (weakly bound outer layers only slightly increase bending strength)	[34]
14	Not specified	Cotton ginning waste and hemp pith (core), fiber fabric (surface)	Not specified	Not specified	110 °C, 24 h	Three layered packaging material	The MCB is light, buoyant, and hydrophilic, and has a soft outer surface with high elasticity. Tensile and compression properties confirm the use of MBC in packaging instead of expanded polystyrene	[35]
15	*Pleurotus pulmonarius*, *P. ostreatus*, *P. salmoneo-stramineus*, *Cyclocybe aegerita* (specified as *A. agrocibe*)	Woodchips of eucalyptus, oak, pine, apple, vine	Autoclaved at 121 °C for 1 h	25 °C, 4–5 weeks	105 °C, 48 h	A foam	The most efficient bonding was observed for *P. ostreatus* grown on apple or vine woodchips	[36]
16	*Pleurotus ostreatus*	Agar, seed, straw, wood, sand and plastic	Autoclaving	22 °C, not specified	Not specified	Spherical fungal assembly elements	Elements made of mycelium may be self-assembling	[37]
17	*Pleurotus ostreatus*, *Fomes fomentarius*	Beech, European oak, pear, spruce, sand or gravel	Autoclaving	25–28 °C, 14–28 days	95 °C	Building component	MBC have advantageous insulating properties, but their stiffness, tensile and compressive strength are not sufficient	[38]
18	Basidiomycetes [21]	Agricultural by-products	Via the process reported by [21]	As [21]	As [21]	Low-density board, 5 levels of densities	Uncompressed MBC boards are low-VOC alternatives to acoustical ceiling tiles in sound shielding applications; Densified MBC boards are alternatives to OSB and MDF. After reaching a density of 0.9 g/cm^3^, the MBC properties do not improve	[40]
19	*Ganoderma lucidum*, *Pleurotus ostreatus*	Cellulose and cellulose/potato-dextrose (PDB)	autoclaved at 120 °C, 15 min	25–30 °C, 70–80% RH, 20 days, agar plug	60 °C, 2 h	Easy-to-grow fibrous mycelium film	The substrate should be homogeneous. The addition of PDB to the substrate increases stiffness of mycelium-based composites	[41]
20	*Irpex lacteus*	Sawdust pulp (*Betula neoalaskana*), millet grain, wheat bran, natural fiber, calcium sulfate	pasteurization	14–42 days	60 °C for 24 h	foam	Densely packed MBC samples have comparable, elastic moduli, compressive strength, and thermal conductivity to the polymeric thermal foams except dry density	[42]
21	Not specified (supplied by Ecovative Design)	Biotex Jute, Biotex Flax, BioMid cellulose plain weave	10% hydrogen peroxide	24 °C, 5 days	82 °C, 12 h and 93 °C, 8 h then pressed (250 °C, 20 min)	Core of sandwich structure	Strength depends on the intensity of mycelium colonization within the skin and the bond between the skin and the core and the substrate. The used fungi preferred flax reinforcement, strength was significantly higher than the jute and cellulose	[43]
22	*Trametes versicolor*, *Daedaleopsis confragosa*, *Ganoderma resinaceum*	Cellulosic fibers: corn stover, kenaf pith, hemp pith	115 °C, 28 min	2 °C, 5 days	Convection oven, 60 °C, 8 h	Improvement of termite resistance	Addition of guayule resin caused maximum MBC repellency to termites; vetiver oil was slightly less effective. Addition of borax was least effective as a termiticide.	[44]
23	Not specified (obtained from Ecovative Design, LLC)	Not specified, Nutrition (calcium and carbohydrate)	Not specified	Not specified	Dried at “elevated temperature” for “several hours”	Pure mycelium	In tension: linear elastic at low strain, and then yields and strain hardening before rupture. In compression: the stress–strain curve has first a linear-elastic form followed by a plateau form with a softened response (similar to open cell foam). In loading and unloading cycles: strain is dependent on hysteresis and progressive stress softening effect (Mullins effect).	[45]
24	*Pleurotus ostreatus*, *Schizophyllum commune*, *Trametes multicolor*	Azolla filiculoides	121 °C, 20 min.	25 °C, 7 days	60 °C	Extractable paste for 3D printing	Applicable for robotic manufacturing of biocomposite structures	[46]
25	*Schizophyllum commune* wild type strain (CBS 341.81) and its derivative Δ*sc3*	Not applivable.	N.A.	30 °C, 1 + 3 + 5 days, in the light or in the dark	dried at room temperature.	Pure mycelium	Mechanical properties of the mycelium of *S. commune* can be changed by inactivating the *sc3* hydrophobin gene. Mechanical properties of wild type mycelium were similar to natural materials, while those of Δsc3 were more similar to thermoplastics	[48]
26	Not specified	Corn stover (three particle size ranges)	Sterilized for 2 h at 15 psi (103.4 kPa)	temp. not specified, 4 + 4 days	100 °C for several hours	Tiles	Increasing supplemental nutrition after a homogenization step increases the mechanical properties of MBC (observed continuity of the mycelium network was greater)	[51]
27	*Trametes* sp., *Schizo-phyllum commune*	Agricultural waste and fruit/vegetable peels	Not specified	25–30 °C, +21 days	Drying above 60 °C	Not compressed, cold and hot compressed boards	Useful for packaging material, furniture, footwear and others	[53]
28	*Oxyporus latermarginatus* (EM26), *Megasporoporia minor* MG65, *Ganoderma resinaceum* GR33	Wheat straw	Autoclaving, 115 °C, 15 min	28 °C, 8 weeks	70 °C	Insulation materials	The choice of fungi species depends on the degradation rate of different substrates. Rapid colonization of the substrate is required because excessive degradation of the substrate leads to weakening of the MBC. MBC shown good thermal performance	[49]
29	*Trametes versicolor*	Glass fines, wheat grains, and rice hulls	121 °C, 15 psi (103.4 kPa), 40 min	25 °C, 50% RH, 12 days	50 °C, 48 h	Fire safe mycelium biocomposites	MBC are safer than the typical construction materials: producing much lower heat release rates, less smoke and CO_2_ and longer time to flashover. The composites with glass fines had the best fire performance.	[50]
30	As [45] (supplied by Ecovative Design, LLC)	Corn stover particles and nutrition (calcium and carbohydrate)	Not specified	25 °C, 4 + 4 days	100 °C, 4 h	Mycelium composites reinforced with agro-waste	The soft elastic response of pure mycelium at small strains (stiffening at larger strains), stress softening effect and hysteresis under cyclic compression were observed	[52]
31	*Trametes multicolor* (*T. ochracea*) (Mycelia BVBAM9915); *Pleurotus ostreatus* (SPOPO Sylvan 195)	Beech sawdust, rapeseed straw, non-woven cotton fiber	Autoclaving	25 °C, 24 days, RH 55–70%, darkness	150 °C, 20 min.	Boards	Straw-based mycelium composites are stiffer and less moisture-resistant than cotton-based	[57]
32	Not specified	Jute, flax, and cellulose textile as outer layers; mycelium-bound agricultural waste with a soy-based bioresin as cores	As in [139]	As in [139]	As in [139]	Three-layer sandwich-structure	Soy-based bioresin significantly increased the mechanical properties of the MBC.	[58]
33	*Trametes versicolor*, *Polyporus brumalis*	Agricultural by-products (wheat straw, rice hulls, sugarcane bagasse, blackstrap molasses) and agricultural products (wheat grains, malt extract)	Autoclaved at 121 °C for 20 min.	25 °C, 7 days, without light	85 °C, 1 h	Pure mycelium	Mycelium grew slow on rice hull, sugarcane bagasse and wheat straw. Liquid blackstrap molasses accelerates growth, outperforming laboratory malt extracts.	[59]
34	Not specified (white-rot basidiomycete mycelium)	Mixture of spruce, pine, and fir	Not specified	Not specified	Dried at 43 °C	Particleboard	Cellulose nanofibers added to the substrate improved the mechanical properties of MBC by 5%	[60]
35	*Colorius* sp., *Trametes* sp., *Ganoderma* sp.	Vine and apple tree-pruning woodchips with 1% flour and 3% wheat straw	Autoclaved at 100 °C, 1 h	23 °C, 95% RH, 14 days	60 °C, 48 h	Foam	Some disadvantages of the material can be turned into advantages, for example, high water absorption could be beneficial in specific applications.	[61]
36	*Coprinopsis cinerea*, *Pleurotus djamor*	Not applicable	Not applicable	cultured at 28 and 37 °C in the dark, then 25 °C under a 12 h light/12 h dark cycle	Biochemically stopped	Not applicable	Biochemical solution to regulate the fruiting body formation, which may replace heat killing of mycelium	[62]
37	*Fomitopsis pinicola*, *Gloeophyllum sepiarium*, *Laetiporus sulphureus*, *Phaeolus schweinitzii*, *Piptoporus betulinus*, *Pleurotus ostreatus*, *Polyporus arcularius*, *Trametes pubescens*, *T. suaveolens*, *Trichaptum abietinum*	Birch, aspen, spruce, pine, fir sawdust and shavings	Sterilized at 121 °C for 60 min.	23 °C, 21 + 21 days	140 °C, 120 min.	Boards	Polyporus arcularius and Trametes suaveolens and birch wood shavings are the best combination	[63]
38	*Lentinula edodes* LED AJU1, *L. edodes* LED CHI, *L. edodes* LED 96/18	Coconut powder, wheat bran	Autoclaved at 121 °C for 60 min.	25 ± 1 °C, 7 + 23 days, without light	50 °C, 24 h	Test samples	The tested composite is suitable as a packaging material	[64]
39	*Trametes versicolor* (M9912)	Flax dust, flax long, wheat straw dust, wheat straw, hemp fibres and pine wood shavings	Autoclaved at 121 °C for 20 min.	28 °C, 16 days	70 °C, 5–10 h	Thermal insulation	The thermal conductivity and water absorption coefficient of MBC are comparable to rock wool, glass wool and extruded polystyrene. The mechanical performance of the MBC depends more on the fiber arrangement than on the chemical composition of the fibers	[65]
40	*Trametes versicolor*	Spruce wood particles	121 °C, 1.25 kPa, 60 min	30 ± 2 °C, 21 days, without light	60 °C, 8 h	Construction material (samples)	Mycelial bond strength is equivalent to synthetic resin bond strength	[66]
41	*Pycnoporus sanguineus* 14G (MIUCS 778), *Pleurotus albidus* 88F.13 (MIUCS 1586), *Lentinus velutinus* 180H.18 (MIUCS 1196)	Pinus sawdust, wheat bran, agar, calcium carbonate	Autoclaved, 30 min, 1 atm	28 °C, for 10 days and 24 ± 2 °C for 15 days	80 °C, 24 h.	Biofoams	Thermogravimetric profile similar to expanded polystyrene, lower thermal stability, but remaining stable up to 350 °C. The compression strength is 60% greater; MBC are biodegradable	[67]
42	*Ganoderma* sp.	56.3% corn stover, 27% grain spawn, 2.4% maltodextrin, 0.8% calcium sulfate, and 13.5% complex of nutrients and mineral mixture	Not specified	30–35 °C	Dried at 43 °C	Board of pure fungal mycelium	Pure mycelium foams is suitable for acoustic shielding products, especially for low to mid-frequency range noise. The mycelium biofoam is also suitable for fire-resistant layers, shoe textile support foams, clothing, and even scaffolding for medical bio-organs and as substitute of meat	[68]
43	*Ganoderma lucidum*	Cassava bagasse, palm sugar fiber, rice bran	Not specified	Room temperature for 12 days	55–60 °C for about 20 h	Construction board	composition of the raw materials affected the density, swelling thickness, water absorption, MOE and MOR	[69]
44	*Ganoderma lucidum*	Cotton stalk	121 °C for 1 h	25 °C, 65% RH for 7 days	65 °C for 10 h	Pressed block	Properties were significantly improved with the increase of hot-pressing temperature	[70]
45	*Ganoderma boninense*	Polyester resin, epoxy resin	Not applicable	Not applicable	Not applicable	Block of composites mushrooms + resin	Mushrooms above 5% decrease in composite hardness	[75]
46	*Ganoderma lucidum*	Cotton stalk, bran	121 °C for 1 h.	25 °C, 65% RH for 7 days	Hot-pressed at 200 °C for 6 min	The mat of 500 × 300 × 12 mm	Strong natural fibers, such as wood and bamboo, are recommended	[80]
47	*Trametes versicolor*	Hemp shives and hardwood chips	Sterilized	22 ± 2 °C, 70 ± 5% RH	93 °C	Lightweight, thermal insulation materials	The strength, water absorption, and biodegradability of 5 combinations of fungi and substrates were compared.	[78]
48	*Ganoderma lucidum*	Bamboo fiber	Pasteurization	30–35 °C, 21 days	80 °C, 9 h	Boards	Non-structural function in buildings	[72]
49	*Ganoderma lucidum*	Potato dextrose broth, D-glucose, alkali lignin	Autoclaved	27 °C, 28 days, 78% RH, in the dark	50 °C, 15 h	Test samples of pure mycelium or mycelium cellulose composite	All mycelia are more or less hydrophobic	[73]
50	Not specified (obtained from Ecovative Design)	Not specified (obtained from Ecovative Design) + wheat flour	Autoclaved	23 ℃, 6–10 days	95 °C, 4 h	3D printed samples	3D printing with biomass–fungi material is possible	[76]
51	*Pleurotus ostreatus*, *P. citrinopileatus*, *P. eryngii*, *G. lucidum*	An undyed nonwoven fabric mat with a fiber content of 45% recycled jute, 49% cotton, 15% cornstarch	80–90 °C, time not specified	25 °C, 7 days	90 °C, 2 h	Biodegradable footwear	Fungi species and substrate (fabric) affected the density. Higher density causes higher compressive strength.	[74]
52	*Trichoderma asperellum*, *Agaricus bisporus*, *P. ostreatus* (HAMBI FBCC0515), *G. lucidum* (HAMBI FBCC665), *P. ostreatus sajor caju* (HAMBI FBCC471), *P. ostreatus florida* (HAMBI FBCC469), *K. mutabilis* (HAMBI FBCC2164), *F. velutipes* (HAMBI FBCC583	Oat husk 1:1, oat and birch sawdust 1:2, oat straw 1:2, rapeseed cake 4:3	120 °C, 20 min	21 °C, 21 days	98 °C, 5 min	Block	MBC with *Agaricus bisporus* gave high resistance to moisture. Hydromechanical stress factors via dynamic mechanical analysis (DMA) are effective to simulate potential conditions for mycelium composites during expected usage.	[77]
53	*Ganoderma lucidum*	Bamboo culms, chitosan	121 °C, 1 h	25–28 °C, RH 65–80%, 7–28 days and 23 ± 0.5 °C, RH 65–70%, 20 days	Dried in an oven	Extrudable paste	Chitosan with mycelium-enriched bamboo is suitable for building elements with complex shapes	[79]
54	*Ganoderma lucidum*, *Pleurotus ostreatus*, *Auricularia polytricha*	Rubber tree (Hevea brasliensis) sawdust, rice bran, lime powder, diaper core, coffee, banana skin, eggshell, sugarcane	Not specified	Not specified	20 min under a 10 MPa pressure at 160 °C	Board	It is possible to produce formaldehyde free bio-boards from spent mushroom substrate.	[81]
55	*Ganoderma lucidum*, *Pleurotus ostreatus*	Clay, sawdust (mixed wood species), bleached and unbleached cellulose	117–120 °C, 0.8–1 bar, 120 min	24 °C, 80% RH, 14 days	600 °C, 6 h, and 960 °C, 2.5 h	Fired brick	Mycelium enhances tensile strength along the extrusion axis and the connection between the layers	[82]
56	*Ganoderma lucidum*, *Trametes hirsuta*, *Pycnoporus sanguineus*, *Fomes fomentarius*	Beech, spruce	121 °C, 2 × 60 min	25 °C, 21–35 days	80 °C, 22 h	Block	The use of wood chips as a substrate causes a higher density of MBC and increases its strength	[83]
57	*Trametes hirsuta*, *Schizophyllum commune*, *Kuehneromyces mutabilis*, *Bjerkandera adusta*, *Gloeophyllum odoratum*, *Lenzites betulina*, *Xylaria hypoxylon*,* Daedalopsis configrosa*, *Coprinellus micaceus*	Sorghum seeds, rapeseed straw	Not specified	25 °C in the dark for 7–10 days + 28 days	6 min at 130 °C with 28 MPa.	Small cylinder	There is a correlation between the extent of colonization and the strength of the material	[1]
58	*Pleurotus ostreatus*	Wood (not specified sawdust)	121 °C, 15 min, sawdust was chemically sterilized	25 °C, 5 days and 24–27 °C, RH 80%, 8–10 days	Not specified	Cylinder	The mycelium biocomposite could substitute expanded polystyrene (EPS)	[84]
59	*Fomes fomentarius*	Fungus fruit body	Not applicable	Not specified	Not applied	Test samples	The fruit bodies of bracket fungi show surprising recovery properties in the wet state	[85]
60	*Trametes versicolor* M9921, *Ganoderma resinaceum* M9726	Hemp hurds, beechwood sawdust	121 °C, 20 min	26 °C, in darkness, 9 days + 22 days	125 °C, 10 h	Compressed board	Producing complex shapes with mycelium materials at the architectural scale is possible	[86]
61	*Ganoderma applanatum*, *Fomes fomentarius*, *Agaricus bisporus*, *Trametes versicolor*	Bleached softwood Kraft fibers, Hemp fibers	165 °C, 75 min and chemically washed	Not specified	Not specified	Test samples	*G. applanatum*, *F. fomentarius*, *A. bisporus*, *T. versicolor* are applicable for blending with cellulose fibers	[87]
62	*Pleurotus ostreatus*	Coir-pith and wood (not specified sawdust)	120 °C, 15 psi (103.4 kPa), 15 min	27 °C, RH 80%, 4 + 14 days	140 °C, 20 min	Board	The mycelium biocomposite could substitute EPS in packaging application	[88]
63	*Ganoderma lucidum*	Wheat straws (90%), polypropylene with bacterial spores (10%)	70% ethanol, rinsed in sterilize water, UV radiation for 10 min	30 °C, 30–35 days	80 °C, for 5 to 10 h	Board	The fungal biocomposite presented similar compressive strength and improved thermal insulation capacity compared to polystyrene	[89]
64	*Pleurotus ostreatus*	Hemp, rice straw, lacquer tree wood chips, and oak wood chips	121 °C, 90 min	20 °C, 65% RH, no light, 7 + 25 days	Not specified	Mycelium composite panels	There is a difference in water absorption rates of the different substrates	[90]
65	*Pleurotus ostreatus*	Sugarcane bagasse, sawdust, rice husk, calcium carbonate, rice bran	Autoclaved at 121 °C for 15 min	25 °C, dark, 7 + 11 days,	100 °C, 24 h	Amorphic biofoam	*P. ostreatus* grows best on rice husk and poorly on sawdust and sugarcane bagasse	[91]
66	*Pycnoporus sanguineus*	Coconut powder, with 30% wheat bran	24, 48 and 72 h at 50, 60 and 70 °C	20 + 13 days	120 °C, 1 atm	Test samples (cubes)	The time and temperature of drying affect the physical properties and microstructure of the biocomposite	[92]
67	*Ganoderma resinaceum*	Hemp shives, soybean hulls	Not specified	22 °C, dark, 7 days	Not applied	Block	There are changes in electrical spiking activity of mycelium bound composites in response to applied heavy loads	[93]
68	*Pleurotus ostreatus*, *F. oxysporum*	Sodium silicate	120 °C, 15 min	24 ± 1 °C		Pure mycelium samples	Adding 3% Si to thenutrient media for *F. oxysporum* increased its thermal stability. The fibers produced by *P. ostreatus* compared with the fibers produced by *F. oxysporum* and improved thermal stability (higher decomposition temperature, lower degradation rate, and higher residual weight)	[56,94]
69	*Basidiomycete (biomass–fungi material (“Grow-It-Yourself”) obtained from Ecovative Design)*	Psyllium husk powder, wheat flour	Not specified	23 °C, 3–5 days	Drying during 3D printing	Pasta to 3D printing	The ratio of psyllium husk powder to water from 1:40 to 2:40 improved 3D print quality	[95]
70	*Pleurotus ostreatus, Oudemansiella radicata, Acremonium* sp.	Cotton stalk, wheat bran	120 °C, 120 kPa, 2 h,	24 ± 1 °C, 28–37 days, RH = 50%	24 °C, 72 h	Block	All tested MBCs presented lower thermal stability but higher residue mass compared to expanded polystyrene. The MBCs proposed in the article could be used as lightweight backfill materials	[96]
71	*Fomes fomentarius*	Hemp shives, rapeseed straw, poplar wood chips, rye grain	Not specified	24 °C, 7 + 7+ 12 + 7 days	70 °C	Brick	The LCA analysis shows an improvement in most impact categories compared to typical building bricks	[97]
72	*Ganoderma lucidum* (M9720)	Empty Fruit Bunch (EFB) fibers, sawdust (Albizia chinensis), wheat bran	120 °C, 60 min	28 °C, 14 days	70 °C, 48 h	Board	The coating is able to retain the material strength over the weathering period in all the loading scenarios	[98]
73	*Pleurotus**ostreatus* (FBCC0515), *T. hirsuta* (FBCC1239)	Softwood shavings, oat bran	30 min. at 120 °C	Growth at 27 °C for 24–27 days, stored at 5 °C for 23 days	at 60, 90, or 120 °C for 3 h	Test samples (beams)	The structure of mycelium more significantly affects the physical characteristics of the mycelium composites than fungal decay modes	[99]
74	*Ganoderma lucidum*	Cellulose fiber, rapeseed bagasse	40 min. at 121 °C	30 °C, 58% RH, 21 + 7 days	Not specified	Foam (wall insulation)	Rapeseed bagasse substrate performed the best in thermal conductivity with the lowest density and good dimension stability, close to conventional EPS polymer	[100]
75	*Trametes versicolor*, (M9912-5LSR-2 O447A) *Ganoderma resinaceum* (M9726)	Beechwood, hemp fiber	20 min. at 121 °C	28 °C for 16 days	70 °C, 5–10 h	Composite and pure mycelium test samples	A method for the disintegration of the mycelium based material was established	[101]
76	*Ganoderma lucidum*	Hemp fibers, hemp hurds, pine wood sawdust, Silvergrass (Miscanthus) shavings	60 min at 121 °C	26–28 °C, 70–80% RH for 14 days	60–70 °C for 2–3 days	Boards with wood reinforcement	The dense boards reinforced with one low-density lattice are the most promising	[102]
77	*Pleurotus ostreatus*	Wood (not specified), hemp fibers	Pasteurization	20–25 °C for 21 days		Prototype furniture made of rattan frame and hemp sheet, jute sheet, hemp rope	The necessity to stop the growth process is the main limitation in the manufacturing on an architectural scale	[103]
78	*Trametes hirsuta*	Cellulose pulp	45 min at 1.5 atm and 121 °C	28 °C for 14 days	Drying	Test samples	A fungal mycelium appears in place of the cellulose microfibrils, but the size of the hyphae differs by an order of magnitude from the size of the cellulose microfibrils.	[104]
79	*Pleurotus ostreatus*	Oak sawdust, Wheat straw, Wheat flour	40 min. at 121 °C	Growth in the bags (14, 21, 28 days) + growth in the formwork (14, 21, 28 days)	2 days at 92 °C	Test samples (cubes)	Substrate mixtures with more sawdust content are harder than straw-based substrate mixtures	[105]
80	*Pleurotus ostreatus*, *Ganoderma lucidum*	beech sawdust, oak sawdust, bleached cellulose pulp, shredded cardboard, shredded newspaper, cotton fibers, soy silk fibers, wheat bran, wheat straw, burlap, clay, and sand	45 min at 121 °C	22–24 °C for 20 + 5 days	Dehydration at 40 °C	Test samples (bricks and beams)	Using a mycelium strain that is more resistant to the water uptake is not sufficient. Hygroscopicity of MBC is highly dependent on the type of substrate used	[106]
81	*Trametes versicolor* (M9912)	Hemp fibers, montmorillonite clay	121 °C for 20 min.	26 °C, 60% RH for 5 + 12 + days	70 °C for 10 h	15 mm board	The nanoclay does not significantly affect the bending behavior	[107]
82	*Fomes fomentarius* (GaG41)	Hemp shives, rapeseed straw	Autoclaving	25 °C for 7 + 14 days	60 °C for 2 days	Test samples	The impact of particle size on compression behavior was more profound for large rapeseed straw particles	[108]
83	*Ganoderma lucidum*	Primary: 11% mycelium spawn, 56% paper pulp, 1% xanthan gum and 32% water by weight. Secondary: sand, gravel, wood chips	Sterilized	The inoculated paper pulp was 3D printed, then the remaining space was filled by a supporting material	Drying	In a mold of 150 × 90 × 90 mm	A multi-material process of fabricating with MBC is required	[109]
84	*Trametes versicolor*, *Ganoderma sessile*, *Trametes multicolor*	Wood (eucalyptus, vine, apple, pine, oak)	121 °C, 1 h	25 °C, 4–5 weeks	105 °C, 48 h	Dense bio composites with low water absorbance and high mechanical properties	Results indicate a correlation between fungi species, substrate, and growth protocol on final MBC characteristics (density, water absorbency, and the compressive strength)	[110]
85	*Ganoderma resinaceum* (GA1M)	Rose flowers and lavender straw	Sterilized	28 °C and 220 rpm for 7 days + 25 °C, 95% RH, for 7 days	60 °C for 8 h	Blocks 40 × 40 × 40 mm	Outer mycelium layer, fibrous internal microporous structure and integrity are appropriate	[111]
86	*Ganoderma lucidum* (M9726)	The 0.2–1.25 mm beechwood sawdust mixed with psyllium husk (*Plantago indica*), corn starch, xanthan gum, paper cellulose, guar gum, and locust bean gum	Autoclaved separately for 20 min at 121 °C (corn starch was heated to 100 °C for 40 min)	26 °C and 60% RH, for 10 days	70 °C for 5 h	3D printed substrate in form of cylinder specimens (*h* = 38 mm, *d* = 100 mm)	The mycelium mitigates crack formation during printing. The core of the extrudable filament was not colonized sufficiently. To 3D print with living materials a dynamic adjustment of nozzle height during printing by scanning the previous layer and control of the deposition is needed.	[112]
87	*Trametes versicolor*	Yellow birch wood particles	Steam-sterilized at 121 °C for 60 min	7-day preincubation, incubated at 28 °C, 80% RH for 8 days, melted and incubated at 28 °C, 80% RH for up to 30 days	Oven-dried for 48 h at 50 °C and hot-pressed at 180 °C for 8 min	Foams, samples with varied dimensions	In the low-density foam, the mycelia bind the particles together, with little impact on the mechanical properties. In hot-pressed panels, the mycelia strengthen the material as a network of hyphae and act as an adhesive.	[113]
